# Creating prognostic systems for cancer patients: A demonstration using breast cancer

**DOI:** 10.1002/cam4.1629

**Published:** 2018-07-02

**Authors:** Mathew T. Hueman, Huan Wang, Charles Q. Yang, Li Sheng, Donald E. Henson, Arnold M. Schwartz, Dechang Chen

**Affiliations:** ^1^ Department of Surgical Oncology John P. Murtha Cancer Center Walter Reed National Military Medical Center Bethesda MD USA; ^2^ Department of Biostatistics The George Washington University Washington DC USA; ^3^ Department of Surgery Walter Reed National Military Medical Center Bethesda MD USA; ^4^ Department of Mathematics Drexel University Philadelphia PA USA; ^5^ Department of Preventive Medicine & Biostatistics F. Edward Hébert School of Medicine Uniformed Services University of the Health Sciences Bethesda MD USA; ^6^ Department of Pathology School of Medicine and Health Sciences The George Washington University Washington DC USA; ^7^ Department of Environmental and Occupational Health Milken Institute School of Public Health The George Washington University Washington DC USA

**Keywords:** breast cancer, cancer staging, C‐index, dendrogram, machine learning, survival

## Abstract

Integrating additional prognostic factors into the tumor, lymph node, metastasis staging system improves the relative stratification of cancer patients and enhances the accuracy in planning their treatment options and predicting clinical outcomes. We describe a novel approach to build prognostic systems for cancer patients that can admit any number of prognostic factors. In the approach, an unsupervised learning algorithm was used to create dendrograms and the C‐index was used to cut dendrograms to generate prognostic groups. Breast cancer data from the Surveillance, Epidemiology, and End Results Program of the National Cancer Institute were used for demonstration. Two relative prognostic systems were created for breast cancer. One system (7 prognostic groups with C‐index = 0.7295) was based on tumor size, regional lymph nodes, and no distant metastasis. The other system (7 prognostic groups with C‐index = 0.7458) was based on tumor size, regional lymph nodes, no distant metastasis, grade, estrogen receptor, progesterone receptor, and age. The dendrograms showed a relationship between survival and prognostic factors. The proposed approach is able to create prognostic systems that have a good accuracy in survival prediction and provide a manageable number of prognostic groups. The prognostic systems have the potential to permit a thorough database analysis of all information relevant to decision‐making in patient management and prognosis.

## 1 INTRODUCTION

The tumor, lymph node, metastasis (TNM) staging system has undergone 8 revisions since first publication of the Cancer Staging Manual in 1976. These revisions have been considered vital in addressing improvements in cancer patient management. The improved modifications have occurred during the accumulation of abundant pathologic and molecular data to expand the understanding of the biology and clinical behavior of cancer. There is a critical need to integrate additional factors into the TNM for a more accurate prediction of patient outcomes. For instance, in the AJCC Cancer Staging Manual (8th edition),^1^ the stage groups of invasive carcinoma of the breast now include grade (G), estrogen receptor (ER), progesterone receptor (PR), and human epidermal growth factor receptor‐2 (HER2), in addition to the traditional primary tumor (T), regional lymph nodes (N), and distant metastasis (M). Incorporating additional factors into the TNM requires mathematical and statistical models. In this report, we describe a novel machine learning‐based approach to build prognostic systems for cancer. In this approach, the Ensemble Algorithm for Clustering Cancer Data (EACCD)^2^, ^3^, ^4^ was used to cluster patients according to survival. The primary output from running the EACCD is a dendrogram that shows how survival varies as levels (categories) of prognostic factors change. Cutting the dendrogram according to the C‐index^5^ divides the patients into various groups. These groups, defined by aggregations of the selected prognostic factors, are analogous to the stage groups in cancer patient staging. We demonstrate applications of the proposed approach to breast cancer.

## 2 MATERIALS AND METHODS

### 2.1 Data

Female breast cancer data were obtained from the Surveillance, Epidemiology, and End Results (SEER) Program of the National Cancer Institute.^6^ In addition to survival and breast cancer‐specific censoring indicator, the factors considered include T, N, M, G, ER, PR, and age (A). Selection of cases, data management, and specifics about factors are described as follows.

SEER initiated the collection of ER and PR in 1990 and T, N, M experienced a major change from earlier Extent of Disease scheme to Collaborative Stage scheme in 2004. Therefore, cases were selected with the year of diagnosis 1990‐2003, involving 9 registries for 1990‐1991, 13 registries for 1992‐1999, and 18 registries for 2000‐2003. This selection also ensured an 11‐year follow‐up to 2014, based on the information released by SEER in 2017.

The present study examines only those cases which were nonmetastatic to distant sites, designated as M0 in the TNM staging. For the histologic grade, grade III and grade IV were combined into a single category to reduce observational variations, and consequently, only 3 grades (low, moderate, and high) were considered. For ER and PR, only “+” and “−” categories were used in this report. Breast cases for ages less than 20 years were not included in the analysis. Cases with age of diagnosis equal to or larger than 20 years were stratified into 2 groups: 20‐50 and 51+. Age 50 was chosen as the cut‐off point because of its wide use in the literature such as helping roughly stratify premenopausal patients from postmenopausal patients.

Table 1 shows the levels (categories) of the factors used in this report. In the table, the definitions of T, N, and M are from Adjusted AJCC 6th ed. T, N, M, and Stage in SEER,^7^ and those of ER and PR are from the SEER Research Data Record Description.^8^ The levels G1 and G2 in the table equal grade I and grade II, respectively, as defined in the SEER Research Data Record Description. The level G3 is either grade III or grade IV. Note that there are no recorded cases with “T0” in the data and thus the definition of “T0” is omitted.

**Table 1:** Definitions of T, N, M, G, ER, PR, and A for SEER breast cancer data diagnosed 1990‐2003

	Categories	Criteria
Tumor size (T)	Tis	Carcinoma in situ
	T1	Tumor 2 cm or less in greatest dimension
	T2	Tumor more than 2 cm but not more than 5 cm in greatest dimension
	T3	Tumor more than 5 cm in greatest dimension
	T4	Tumor of any size with direct extension to (a) chest wall or (b) skin, only as described below T4a Extension to chest wall, not including pectoralis muscle T4b Edema (including peau d'orange) or ulceration of the skin of the breast, or satellite skin nodules confined to the same breast T4c Both T4a and T4b T4d Inflammatory carcinoma
Regional positive lymph nodes (N)	N0	No regional lymph node metastasis histologically
	N1	Metastasis in 1 to 3 axillary lymph nodes, and/or in internal mammary nodes with microscopic disease detected by sentinel lymph node dissection but not clinically apparent
	N2	Metastasis in 4 to 9 axillary lymph nodes, or in clinically apparent internal mammary lymph nodes in the absence of axillary lymph node metastasis
	N3	Metastasis in 10 or more axillary lymph nodes, or in infraclavicular lymph nodes, or in clinically apparent ipsilateral internal mammary lymph nodes in the presence of 1 or more positive axillary lymph nodes; or in more than 3 axillary lymph nodes with clinically negative microscopic metastasis in internal mammary lymph nodes; or in ipsilateral supraclavicular lymph nodes
Distant metastasis (M)	M0	No distant metastasis
	M1	Distant metastasis
Histological grade (G)	G1	Well differentiated
	G2	Moderately differentiated
	G3	Poorly differentiated or undifferentiated
Estrogen receptor expression (ER)	ER+	Cancer cells can receive signals from estrogen
	ER−	Cancer cells cannot receive signals from estrogen
Progesterone receptor expression (PR)	PR+	Cancer cells can receive signals from progesterone
	PR−	Cancer cells cannot receive signals from progesterone
Age at diagnosis (A)	A1	Age at diagnosis ranging from 20 to 50 in years
	A2	Age at diagnosis equal to or larger than 51 in years

The characteristics of patients are described by their associated combination of prognostic factors. A combination is a subset of the data that corresponds to one level of each selected factor. For example, T1 and N0 produce a combination, denoted by T1N0, which represents a subset of patients whose tumor size is T1 and lymph node status is N0. As in T1N0, we use notations of levels of factors to denote combinations in this report.

Two datasets were used for our study. Dataset 1 consisted of cases with a complete record of survival, censoring status, year of diagnosis, T, N, and M (=M0). Due to the use of statistical techniques in our algorithm, we required that the minimum number of breast cancer cases in each combination of T, N, and M in Dataset 1 be 100. (The use of the cut‐off number 100 assures that each combination contains a sufficient number of patients and a certain number of deaths. A smaller cut‐off number serving the same purpose can also be used.) Dataset 1 contained 314391 cases, comprising 17 combinations in terms of T, N, and M. Dataset 2 consisted of cases (black and white women) with a complete record of survival, censoring status, year of diagnosis, T, N, M, G, ER, PR, and A. We also required that the number of cases in each combination of T, N, M, G, ER, PR, and A be at least 100. Dataset 2 contained 199822 cases, comprising 165 combinations on the basis of T, N, M, G, ER, PR, and A.

### 2.2 EACCD

The EACCD is an unsupervised learning algorithm. The computer program algorithm analyzes combinations according to survival and thereby generates clusters of survival outcomes. In the analysis of this paper, we use one modified version of the EACCD. The algorithm consists of 3 steps: (1) computing initial dissimilarities which compares pairwise combinations in terms of survival; (2) computing learned dissimilarities which teaches the program to learn the data‐driven difference in survival between combinations; and (3) performing hierarchical cluster analysis which creates the dendrogram visualizing a relationship between survival and prognostic factors. The details of these steps are given as follows.

We define the initial dissimilarity between 2 combinations using the ratio of hazard functions.^9^ Such a ratio is an effect size (independent of the sample size) that measures the magnitude of the difference (with respect to survival) between 2 combinations. Assuming the proportional hazards model^10^ holds for the populations represented by combinations, the ratio of the hazard rates corresponding to 2 combinations is a constant. In general, there are 2 different ratios (one is the reciprocal of the other). The larger ratio is used as the initial dissimilarity between 2 combinations.

We compute learned dissimilarities between 2 combinations by employing the initial dissimilarities and the 2‐phase Partitioning Around Medoids (PAM) algorithm.^11^ Specifically, we use the initial dissimilarities and PAM to partition available combinations into *k* clusters, where *k* ranges from 1 to *n* with *n* denoting the total number of combinations. For each *k*, we define δ_*k*_(*i*, *j*) = 1 if combinations *i* and *j* are not assigned into the same cluster and δ_*k*_(*i*, *j*) = 0 otherwise. The learned dissimilarity between combinations *i* and *j* is defined as the ratio $\sum_{k=1}^{n}\delta_{k}\left(i,\;j\right)$ to *n*, which is simply the percentage of the times *i* and *j* are not placed into the same cluster by the PAM algorithm. The learned dissimilarities, which are between 0 and 1, are more data‐driven than the initial dissimilarities.

With the learned dissimilarities, the combinations are then clustered by applying the complete linkage method.^12^ The primary output of this hierarchical cluster analysis is a dendrogram that provides a graphical summary of patients’ survival based on the levels of prognostic factors.

### 2.3 Prognostic systems

Given any number of prognostic factors, the EACCD method generates a dendrogram that displays all possible combinations. Given the complexity of the dendrogram, combinations are then partitioned so that those with “similar” survival are grouped together. Each group amalgamates a cohort of distinct combinations. The resultant cohort stratification produces prognostic group assignments which serve the same role as a staging system.

Partitioning combinations exhibited in a dendrogram can be accomplished by horizontally cutting the dendrogram at a specific level of dissimilarity. With available groups of combinations that are obtained by cutting the dendrogram, the C‐index can be computed to indicate a statistical predictive accuracy. The C‐index is an estimate of the probability that a patient who experienced an event (eg, death) in an earlier time had a shorter predicted time than a patient who experienced the event in a later time. A higher C‐index implies a higher accuracy in survival prediction. In general, the curve of the C‐index versus the number of groups increases for relatively small numbers of groups and then quickly plateaus as more groups are generated. The optimal level of stratifying the dendrogram is achieved when the minimum number, (denoted by n*), of groups yields the maximum value of the C‐index.

We define a prognostic system as a collection of the dendrogram, the (maximum) C‐index corresponding to cutting, the group assignment, and the survival curves for groups. We call each group in a prognostic system a prognostic group. Throughout the paper, the breast cancer specific survival curves are estimated by the Kaplan‐Meier method.^13^

## 3 RESULTS

In this section, we presented a prognostic system of breast cancer for T, N, and M and compare it with the historical AJCC 6th edition staging system.^14^ We then created a prognostic system based on T, N, M, G, ER, PR, and A.

### 3.1 Prognostic system on the basis of T, N, and M

**Figure 1:**
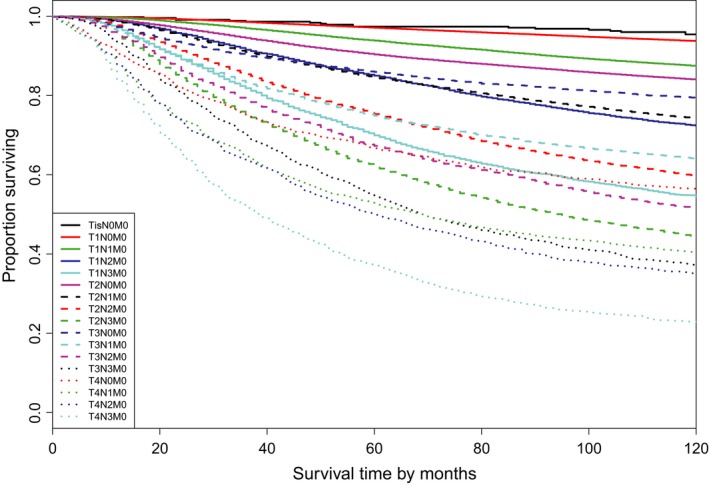
Breast cancer‐specific survival of 17 combinations of SEER breast cancer patients diagnosed 1990‐2003

**Figure 2:**
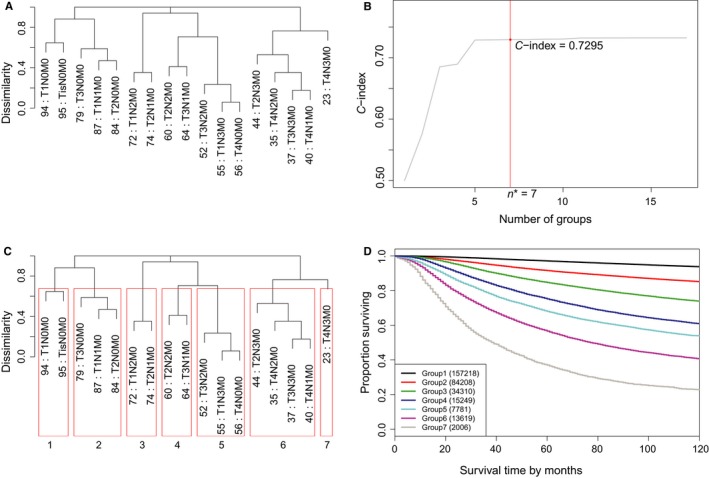
Generation of the prognostic system on the basis of T, N, and M using the SEER breast cancer patients diagnosed 1990‐2003. (A) Dendrogram for 12 combinations according to T, N, and M. A 10‐year survival rate in percentage is given beneath each combination. (B) C‐index curve and n*(=7). (C) Cutting the dendrogram according to n*. (D) Breast cancer‐specific survival of 7 prognostic groups

We applied EACCD to Dataset 1 to generate a prognostic system of breast cancer based only on T, N, and M. The data consisted of 17 combinations of T, N, and M with each individual combination having a minimum of 100 cases. The survival curves of these 17 combinations are presented in Figure 1. The dendrogram from running EACCD is shown in Figure 2A, which displays the relationship (with respect to survival) among combinations. The C‐index curve is given in Figure 2B, which indicates n* = 7 with a C‐index of 0.7295. (In Figure 2B, the values of C‐index are similar when the number of groups is close to 7. We chose n*=7 because we planned to compare the generated system with the AJCC grouping that has 7 stage groups.) Cutting the dendrogram using n* (Figure 2C) leads to 7 prognostic groups listed in the first two columns of Table 2. The survival curves of these prognostic groups are presented in Figure 2D. The dendrogram with cutting in Figure 2C, the 7 prognostic groups in Table 2, and the survival curves in Figure 2D define a prognostic system (C‐index = 0.7295) for the breast cancer in light of T, N, and M.

**Table 2:** EACCD and AJCC grouping of the SEER breast cancer patients diagnosed 1990‐2003

EACCD		AJCC	
Group 1	TisN0M0 T1N0M0	Stage 0	TisN0M0
Group 2	T1N1M0	Stage I	T1N0M0
	T2N0M0		
	T3N0M0		
Group 3	T1N2M0	Stage IIA	T1N1M0
	T2N1M0		T2N0M0
Group 4	T2N2M0	Stage IIB	T2N1M0
	T3N1M0		T3N0M0
Group 5	T1N3M0	Stage IIIA	T1N2M0
	T3N2M0		T2N2M0
	T4N0M0		T3N1M0
			T3N2M0
Group 6	T2N3M0	Stage IIIB	T4N0M0
	T3N3M0		T4N1M0
	T4N1M0		T4N2M0
	T4N2M0		
Group 7	T4N3M0	Stage IIIC	T1N3M0
			T2N3M0
			T3N3M0
			T4N3M0

**Figure 3:**
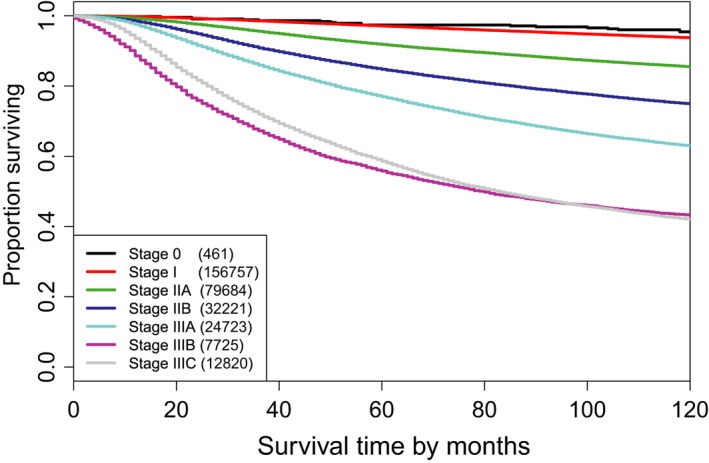
Breast cancer‐specific survival of 7 AJCC stage groups defined in Table 2

For a comparison, the AJCC Cancer Staging Manual (6th edition) partitions Dataset 1 into 7 stage groups shown in the third and fourth columns of Table 2 and Figure 3. Note that the breast cancer anatomic staging system in the AJCC 6th edition is the same as the one in the 7th or 8th edition except that the latter two versions split stage I into stage IA and stage IB. However, differentiating stage IA from stage IB requires a category “N1mi” which was not available in SEER until 2004. Therefore, we used the AJCC staging in the 6th edition here. Also note that stage IV in AJCC staging (6th edition) was not included since our data were restricted to M0.

Two direct comparisons between AJCC and EACCD can be made. One involves overlapping. In the AJCC system, the survival curves of stage IIIB and stage IIIC overlap with each other (Figure 3). The survival curve of stage IIIB is lower than the curve of stage IIIC until year 8. The issue of overlapping does not occur with EACCD (Figure 2D). Another comparison is regarding the C‐index. Calculation shows the AJCC system (Table 2 and Figure 3) has a C‐index of 0.7287, which is lower than the C‐index (0.7295) of the system in Figure 2. This implies that the performance of the prognostic system generated by EACCD is at least as good as the AJCC staging system in terms of the accuracy in survival prediction.

### 3.2 Prognostic system on the basis of T, N, M, G, ER, PR, and A

**Figure 4:**
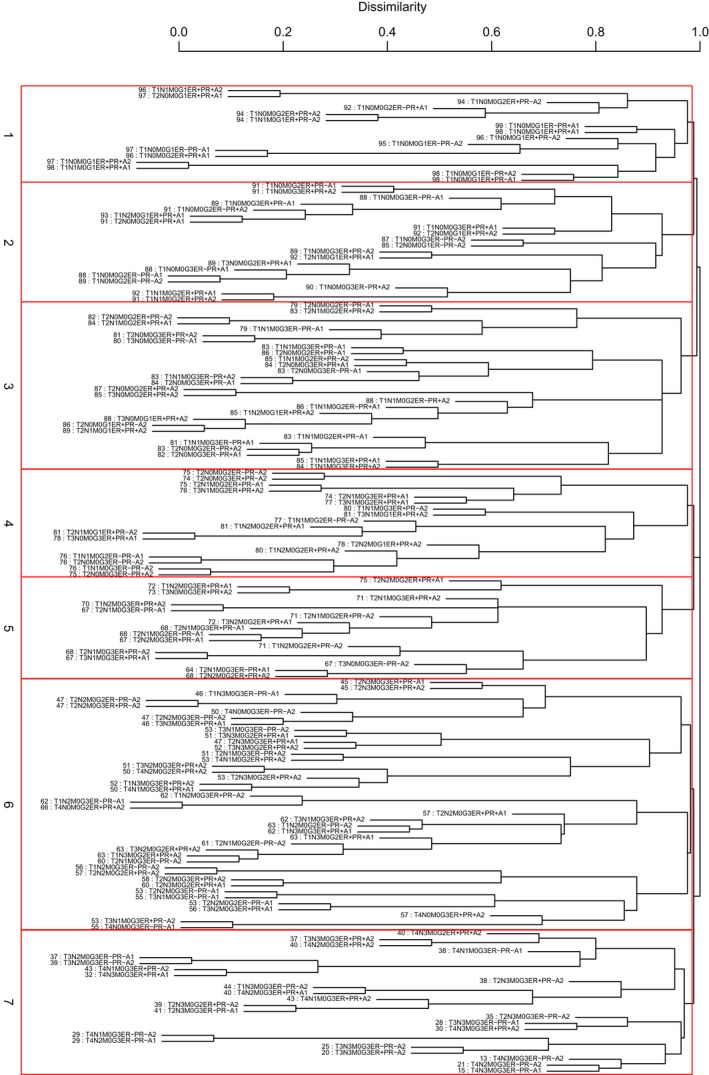
Dendrogram (in black color) for 165 combinations of the SEER breast cancer patients diagnosed 1990‐2003. A 10‐year survival rate in percentage is given beneath each combination. Lines in red color show 7 prognostic groups from cutting the dendrogram

**Figure 5:**
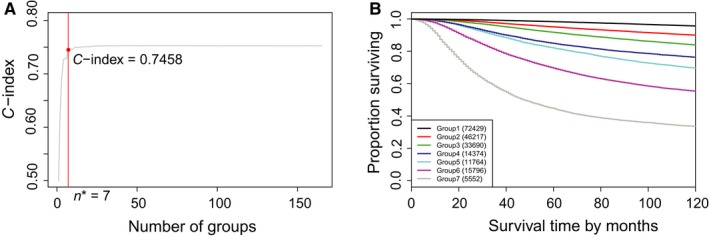
(A) C‐index curve and n*(=7) using the dendrogram in Figure 4. (B) Breast cancer‐specific survival of 7 prognostic groups from cutting the dendrogram in Figure 4 according to n*

We used Dataset 2 to build a prognostic system according to T, N, M, G, ER, PR, and A. The data consist of 165 combinations based on T, N, M, G, ER, PR, and A with each including at least 100 patients. Figures 4 and 5 show a prognostic system with 7 prognostic groups and a C‐index of 0.7458, where a detailed definition of prognostic groups is given in Table 3.

**Table 3:** EACCD grouping of the SEER breast cancer patients diagnosed 1990‐2003

T	N	M	G	ER	PR	A
*Group 1*						
T1	N0	M0	G1	Any ER	Any PR	20+
T1	N0	M0	G2	ER−	PR+	20‐50
T1	N0	M0	G2	ER+	PR−	51+
T1	N0	M0	G2	ER+	PR+	20+
T1	N1	M0	G1	ER+	PR−	51+
T1	N1	M0	G1	ER+	PR+	20+
T2	N0	M0	G1	ER+	PR+	20‐50
*Group 2*						
T1	N0	M0	G2	ER−	PR−	20+
T1	N0	M0	G2	ER−	PR+	51+
T1	N0	M0	G2	ER+	PR−	20‐50
T1	N0	M0	G3	Any ER	Any PR	20+
T1	N1	M0	G2	ER+	PR+	20+
T1	N2	M0	G1	ER+	PR+	20‐50
T2	N0	M0	G1	ER−	PR−	51+
T2	N0	M0	G1	ER+	PR+	51+
T2	N0	M0	G2	ER+	PR+	20‐50
T2	N1	M0	G1	ER+	PR+	20‐50
T3	N0	M0	G2	ER+	PR+	20‐50
*Group 3*						
T1	N1	M0	G2	ER−	PR+	20+
T1	N1	M0	G2	ER+	PR−	20+
T1	N1	M0	G3	ER−	PR−	20‐50
T1	N1	M0	G3	ER−	PR+	20+
T1	N1	M0	G3	ER+	PR−	20‐50
T1	N1	M0	G3	ER+	PR+	20+
T1	N2	M0	G1	ER+	PR+	51+
T2	N0	M0	G1	ER+	PR−	51+
T2	N0	M0	G2	ER−	PR−	20‐50
T2	N0	M0	G2	ER−	PR+	51+
T2	N0	M0	G2	ER+	PR−	20+
T2	N0	M0	G2	ER+	PR+	51+
T2	N0	M0	G3	ER−	Any PR	20‐50
T2	N0	M0	G3	ER+	PR−	20‐50
T2	N0	M0	G3	ER+	PR+	20+
T2	N1	M0	G1	ER+	PR+	51+
T2	N1	M0	G2	ER+	PR+	20+
T3	N0	M0	G1	ER+	PR+	51+
T3	N0	M0	G2	ER+	PR+	51+
T3	N0	M0	G3	ER−	PR−	20‐50
*Group 4*						
T1	N1	M0	G2	ER−	PR−	20+
T1	N1	M0	G3	Any ER	PR−	51+
T1	N2	M0	G2	ER+	PR+	20+
T2	N0	M0	G2	ER−	PR−	51+
T2	N0	M0	G3	ER−	Any PR	51+
T2	N0	M0	G3	ER+	PR−	51+
T2	N1	M0	G1	ER+	PR−	51+
T2	N1	M0	G2	ER+	PR−	20‐50
T2	N1	M0	G3	ER+	PR+	20‐50
T2	N2	M0	G1	ER+	PR+	51+
T3	N0	M0	G3	ER+	PR+	20‐50
T3	N1	M0	G1	ER+	PR+	51+
T3	N1	M0	G2	ER+	PR+	20+
*Group 5*						
T1	N2	M0	G2	ER+	PR−	51+
T1	N2	M0	G3	ER+	PR+	20+
T2	N1	M0	G2	ER−	PR−	20‐50
T2	N1	M0	G2	ER+	PR−	51+
T2	N1	M0	G3	ER−	Any PR	20‐50
T2	N1	M0	G3	ER+	PR−	20+
T2	N1	M0	G3	ER+	PR+	51+
T2	N2	M0	G2	ER+	PR+	20+
T2	N2	M0	G3	ER+	PR−	20‐50
T3	N0	M0	G3	ER−	PR−	51+
T3	N0	M0	G3	ER+	PR+	51+
T3	N1	M0	G3	ER+	PR+	20‐50
T3	N2	M0	G2	ER+	PR+	20‐50
*Group 6*						
T1	N2	M0	G2	ER−	PR−	51+
T1	N2	M0	G3	ER−	PR−	20+
T1	N2	M0	G3	ER+	PR−	51+
T1	N3	M0	G2	ER+	PR+	20+
T1	N3	M0	G3	ER−	PR−	20‐50
T1	N3	M0	G3	ER+	PR+	20+
T2	N1	M0	G2	ER−	PR−	51+
T2	N1	M0	G3	ER−	Any PR	51+
T2	N2	M0	G2	ER−	PR−	20+
T2	N2	M0	G2	ER+	PR−	51+
T2	N2	M0	G3	ER−	PR−	20+
T2	N2	M0	G3	ER+	PR−	51+
T2	N2	M0	G3	ER+	PR+	20+
T2	N3	M0	G2	ER+	PR+	20+
T2	N3	M0	G3	ER+	PR−	20‐50
T2	N3	M0	G3	ER+	PR+	20+
T3	N1	M0	G3	ER−	PR−	20+
T3	N1	M0	G3	ER+	Any PR	51+
T3	N2	M0	G2	ER+	PR+	51+
T3	N2	M0	G3	ER+	PR+	20+
T3	N3	M0	G2	ER+	PR+	20+
T3	N3	M0	G3	ER+	PR+	20‐50
T4	N0	M0	G2	ER+	PR+	51+
T4	N0	M0	G3	ER−	PR−	20+
T4	N0	M0	G3	ER+	PR+	51+
T4	N1	M0	G2	ER+	PR+	51+
T4	N1	M0	G3	ER+	PR+	20‐50
T4	N2	M0	G2	ER+	PR+	51+
*Group 7*						
T1	N3	M0	G3	ER−	PR−	51+
T2	N3	M0	G2	ER+	PR−	51+
T2	N3	M0	G3	ER−	PR−	20+
T2	N3	M0	G3	ER+	PR−	51+
T3	N2	M0	G3	ER−	PR−	20+
T3	N3	M0	G3	ER−	PR−	20+
T3	N3	M0	G3	ER+	Any PR	51+
T4	N1	M0	G3	ER−	PR−	20+
T4	N1	M0	G3	ER+	Any PR	51+
T4	N2	M0	G3	ER−	PR−	20+
T4	N2	M0	G3	ER+	PR+	20+
T4	N3	M0	G2	ER+	PR+	51+
T4	N3	M0	G3	ER−	PR−	20+
T4	N3	M0	G3	ER+	PR+	20+

The C‐index of the system based on T, N, M, G, ER, PR, and A is 0.7458, which is higher than the C‐index (0.7295) of the prognostic system based on T, N, and M. This indicates that adding G, ER, PR, and A into the collection {T, N, M} increases accuracy in survival prediction by 0.7458‐0.7295 = 0.0163.

## 4 DISCUSSION

We described a systematic approach to creating prognostic systems for cancer. The approach contains two major steps: running the EACCD to produce dendrograms and cutting dendrograms to generate prognostic groups according to the C‐index. We demonstrated the approach using the SEER breast cancer data.

In addition to providing prognostic groups analogous to stage groups, a prognostic system (as reported in this study) for breast cancer provides other valuable information. It presents Kaplan‐Meier estimates of breast cancer specific survival for each prognostic group. It provides the C‐index indicating the accuracy of survival prediction. It also supplies a dendrogram showing details on the change of survival rates as factor levels vary.

The biomarker HER2, important for management and prognostic information, was not included in the present study. We focused on data with at least 10 years follow‐up and SEER initiated collection of HER2 results in 2010. Future studies with more robust data entries will include this important biomarker.

The approach to creating prognostic systems described in this paper has several advantages. First, it admits any number of prognostic factors. With a large number of prognostic factors included in the data, the approach can generate a small number of manageable prognostic groups that have approximately the same survival prediction accuracy as the original data. Second, the approach can be readily applied to integrate any prognostic factors including biomarkers into the TNM staging system to predict the outcome of patients. Third, the survival curves of prognostic groups, derived from the approach, do not show crossover in general. This facilitates the use of the prognostic systems by clinicians and researchers. Fourth, the dendrogram from the approach provides details on the change of survival rates as levels of factor vary. This can be useful for accurate clinical counseling and aid discussions regarding mortality, as well as open other avenues of epidemiologic research for patient survival. Fifth, the approach can be applied to any survival data for any noncancer disease to produce prognostic systems for the disease under study. Finally, this data‐driven approach applies whenever the data are available, therefore providing timely updates of prognostic groups and their survivals.

In summary, the proposed approach is able to create prognostic systems that have a good accuracy in survival prediction and provide a manageable number of prognostic groups. The prognostic systems have the potential to permit a thorough database analysis of all information relevant to decision‐making in patient management and prognosis.

## DISCLAIMER

The contents, views or opinions expressed in this publication or presentation are those of the authors and do not necessarily reflect official policy or position of Uniformed Services University of the Health Sciences, the Department of Defense (DoD), or Departments of the Army, Navy, or Air Force. Mention of trade names, commercial products, or organizations does not imply endorsement by the U.S. Government.

## CONFLICT OF INTEREST

There are no conflict of interest disclosures from any authors.
